# Macroglossia Due to Symmetrical Lipomatosis of the Tongue: A Rare Presentation of Madelung's Disease—Case Report

**DOI:** 10.1002/ccr3.72199

**Published:** 2026-02-28

**Authors:** Daniela Salamanca‐Ramirez, Daniel Felipe Villa‐Zuluaga, Diego Alejandro Ardila‐Torres

**Affiliations:** ^1^ Faculty of Medicine Pontificia Universidad Javeriana Bogotá Colombia; ^2^ Faculty of Medicine Universidad Nacional de Colombia Bogotá Colombia; ^3^ Department of General and Head and Neck Surgery Fundación Universitaria de Ciencias de la Salud (FUCS) and Clínica La Colina Bogotá Colombia

**Keywords:** benign symmetrical lipomatosis, Launois–Bensaude syndrome, Madelung's disease, multiple symmetrical lipomatoses

## Abstract

Madelung's disease can present with macroglossia, leading to airway and functional impairment. Early diagnosis and surgical intervention are essential to prevent complications and improve quality of life. This case highlights the importance of recognizing rare presentations for prompt and effective management.

## Graphical abstract

Symmetrical lipomatosis of the tongue is a rare manifestation of Madelung´s disease, characterized by diffuse, non‐encapsulated adipose tissue growth. We present the first reported Latin American case of a 58 year old patient treated with symmetrical bilateral glossectomy which preserved tongue functionality and significantly improved quality of life.

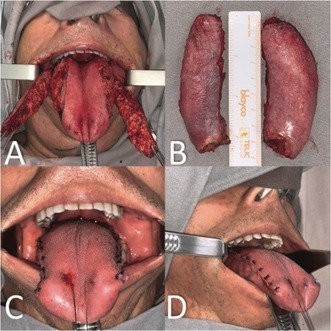

## 1 Introduction

Madelung's disease, also known as Launois–Bensaude syndrome, is a Benign Symmetrical Lipomatosis (BSL) characterized by multiple lipomatous deposits in typical areas such as the neck, shoulders, and back, with uncommon presentations in the scrotum and tongue [1]. It is more common in men, with a male‐to‐female ratio ranging from 15:1 to 30:1. BSL predominantly affects individuals between the ages of 40 and 79, with a higher prevalence among populations from Mediterranean regions. The disease is associated with alcohol consumption and metabolic disorders such as dyslipidemia, hypertension, and liver disease [2].

The pathophysiology of Madelung's disease is not fully understood; however, it has been associated with several mechanisms, including alterations in adrenergic receptors, mutations in mitochondrial DNA, and enzymatic defects. These abnormalities may impair adrenergic‐mediated lipolysis and lead to sympathetic denervation of brown adipose tissue, ultimately resulting in adipocyte hypertrophy [3].

Madelung's disease is classified into two main types. Type I is characterized by well‐defined fat deposits in the neck (known as Madelung's collar or “horse collar”), shoulders, supraclavicular region, and proximal upper limbs. These masses may cause clinical complications such as dysphagia or airway compression. In contrast, Type II presents with diffuse fat distribution in the abdomen and thighs, resembling simple obesity [3, 4]. Diagnosis is based on clinical evaluation and imaging studies, including MRI, computed tomography, and ultrasonography [5]. Given that macroglossia is more often linked to malignancy, a differential diagnosis is essential. Benign tumors of the tongue are relatively uncommon, with lymphangioma, cavernous hemangioma, and neurofibromatosis being the most frequently reported causes [6]. Histological examination of Madelung's disease typically reveals nonencapsulated adipose tissue without cellular atypia [1, 4].

This report presents a rare case of macroglossia caused by symmetrical lipomatosis associated with Madelung's disease.

## 2 Case History/Examination

A 58‐year‐old Latin American male was referred to our department with Madelung's disease, phenotype I, presenting with progressive macroglossia over the past 5 years, accompanied by dysarthria, dysphagia, and supine dyspnea. His medical history included bilateral hearing loss, hypertension, vertigo, and prior surgical resection of lipomas in the neck and shoulders. The patient denied alcohol consumption.

## 3 Diagnosis

**FIGURE 1:**
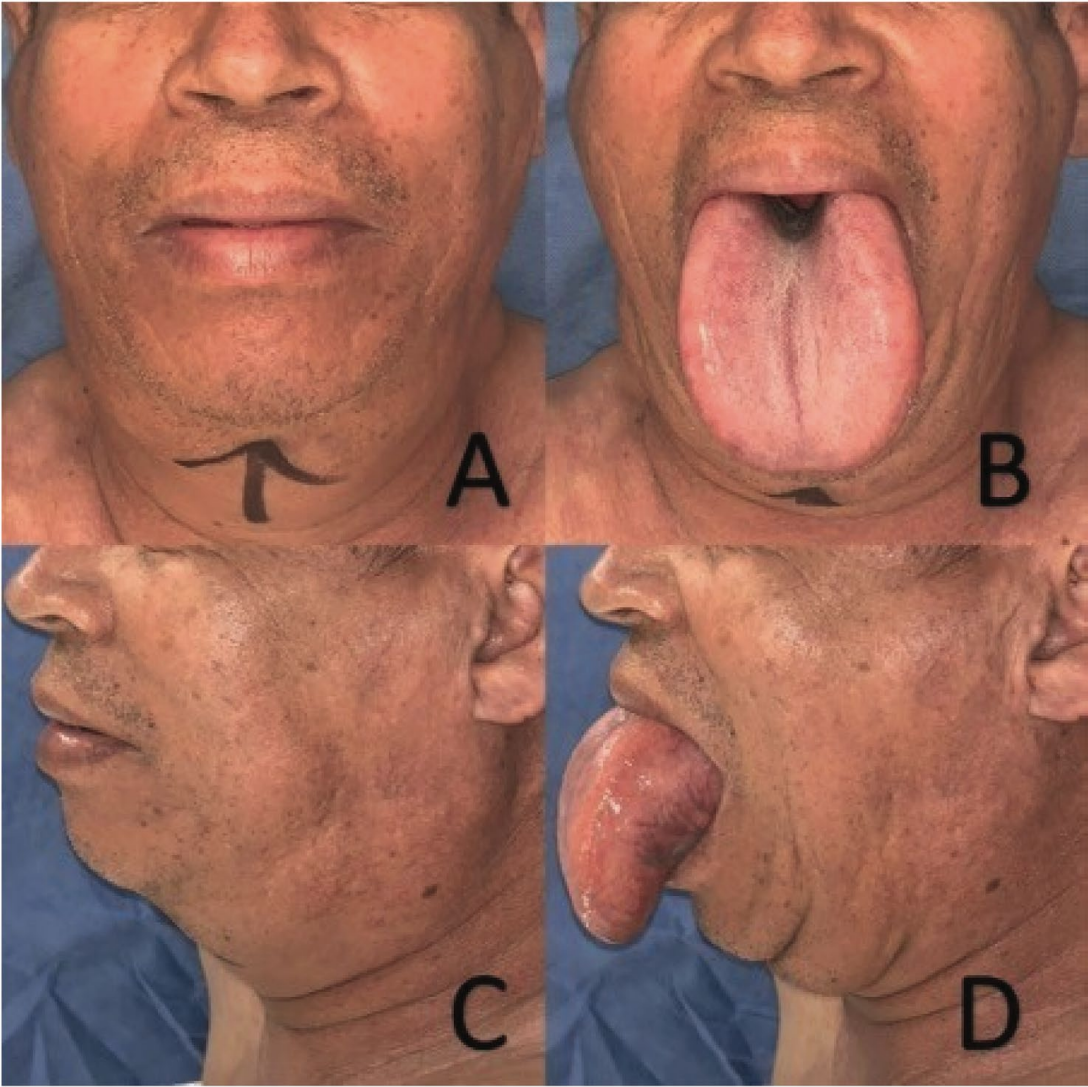
(A) Frontal view of the face showing submental widening. (B) Generalized macroglossia, frontal view. (C) Lateral view of the face. (D) Generalized macroglossia, lateral view.

**FIGURE 2:**
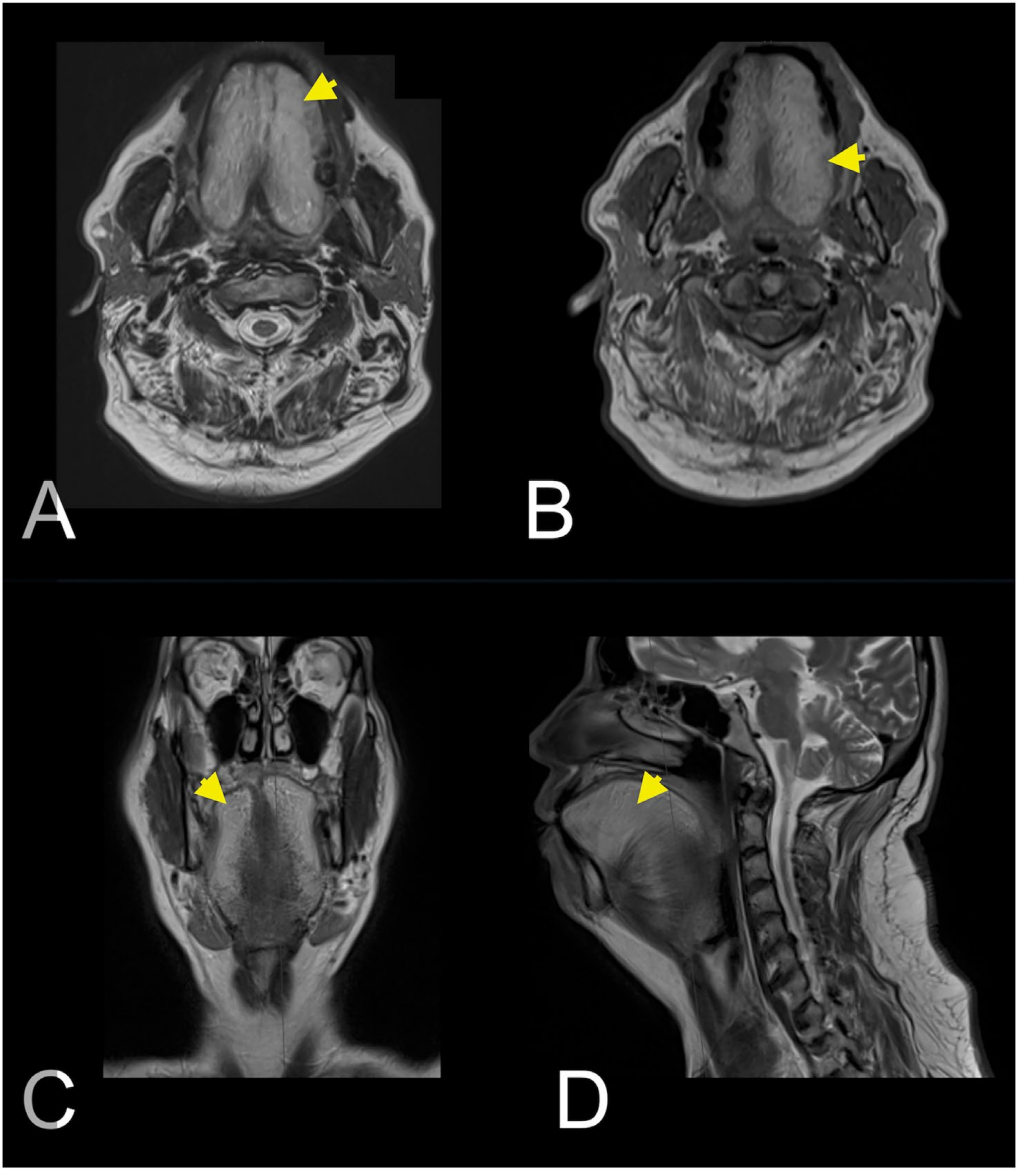
(A) Axial T2‐weighted MR image showing benign symmetrical lipomatosis (BSL) with high signal intensity (arrowheads), typical of adipose tissue. (B) Axial T1‐weighted MR image confirming BSL with a similar high signal intensity pattern. (C) Coronal T2‐weighted MR image showing BSL with high signal intensity (arrowheads). (D) Sagittal T2‐weighted MR image demonstrating BSL with high signal intensity (arrowheads).

Physical examination revealed generalized macroglossia without limitations in oral closure or opening, along with submental widening (Figure 1). MRI revealed a markedly enlarged tongue with hyperintense signals on both T1‐ and T2‐weighted images, consistent with benign symmetrical lipomatosis of the tongue, characteristic of Madelung's disease (Figure 2). Given the characteristics of the lesion and its impact on the patient's quality of life, a symmetrical bilateral glossectomy was performed as the definitive treatment.

## 4 Treatment—Surgical Technique

**FIGURE 3:**
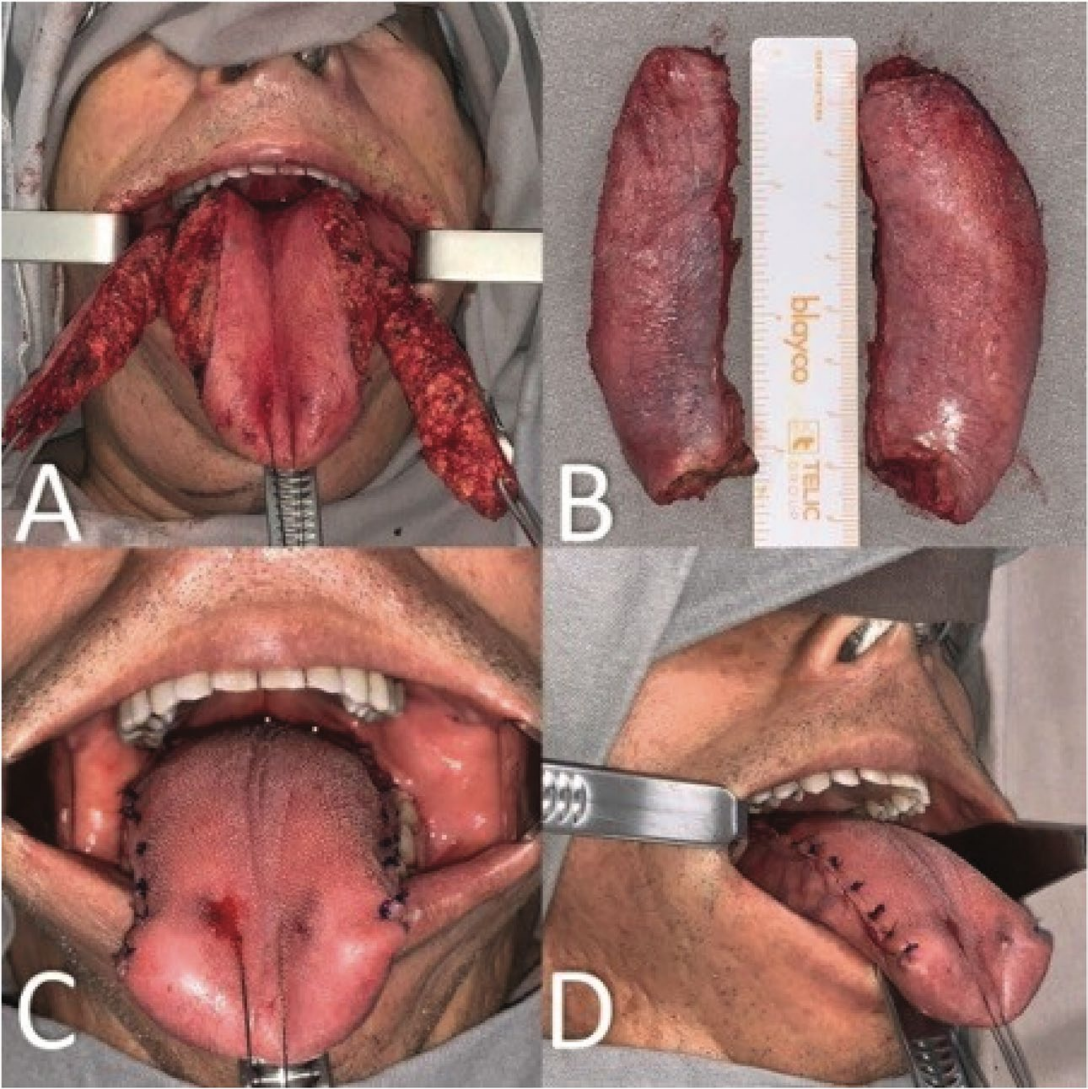
(A) Nonencapsulated lipomatous tissue in the lingual parenchyma. (B) Product of symmetrical bilateral partial glossectomy, each fragment measuring 8.5 x 2 x 3 cm. (C) Frontal view of glossorrhaphy. (D) Lateral view of glossorrhaphy.

The tongue was measured at 15 cm in length, 9 cm in width, and 3 cm in thickness. Preoperative incisions were marked to preserve as many taste buds as possible (Figure 3). Lateral dissection revealed nonencapsulated adipose tissue (Figure 3A). Two fragments (8.5 x 2 x 3 cm each), equivalent to approximately 35% of the tongue volume, were excised (Figure 3B). Vertical mattress sutures with polydioxanone were used for glossorrhaphy to evert edges, prevent dehiscence, and obliterate dead spaces (Figure 3C,D).

**FIGURE 4:**
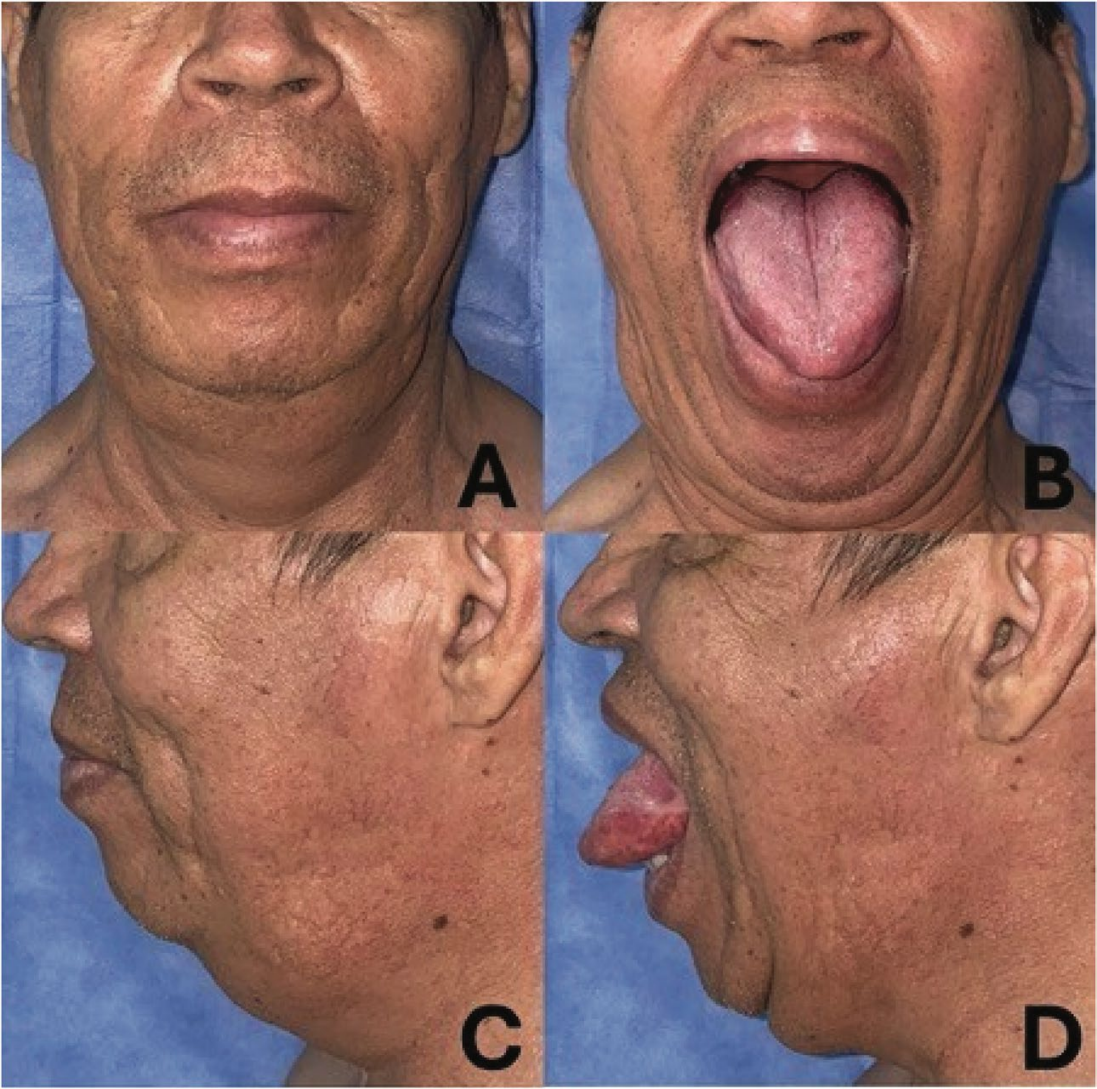
(A) Postoperative frontal view. (B) Frontal view of the postoperative result of symmetrical bilateral partial glossectomy. (C) Postoperative lateral view. (D) Lateral view of the postoperative result of symmetrical bilateral partial glossectomy.

**FIGURE 5:**
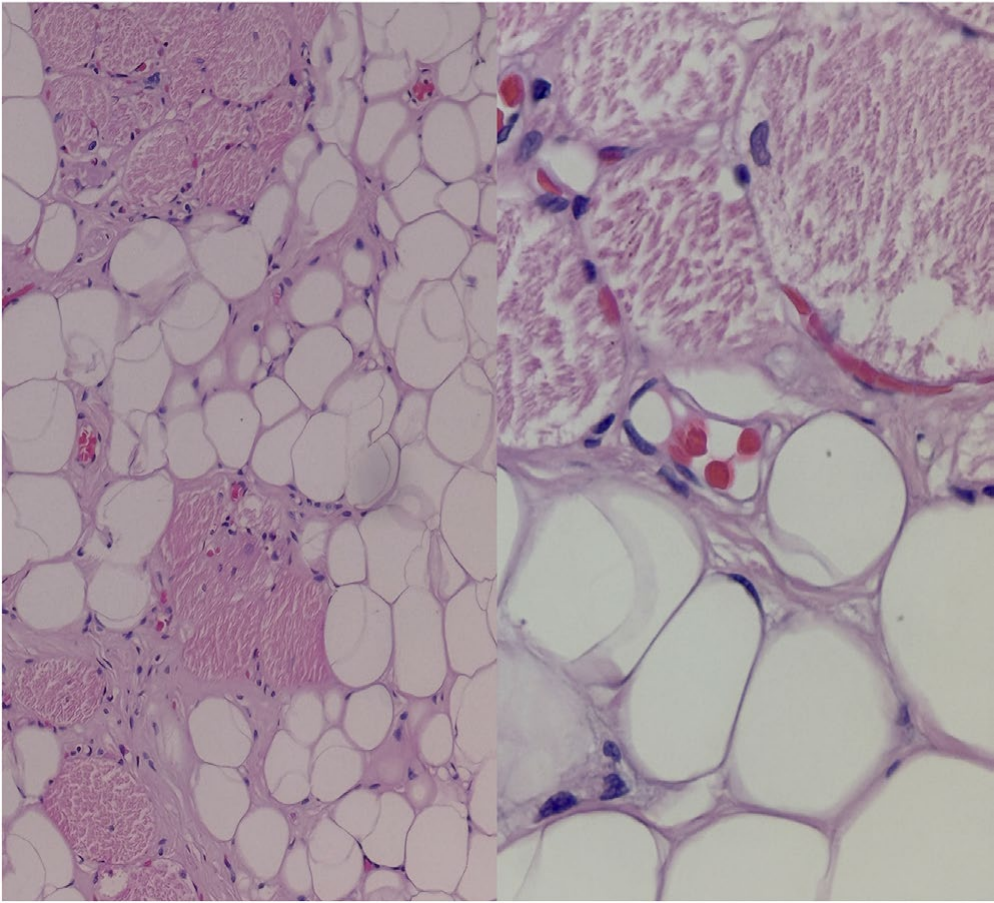
Mature adipose tissue without atypia, arranged in a diffuse growth pattern with interspersed muscle fibers. No lipoblasts or immature stromal cells are identified.

**FIGURE 6:**
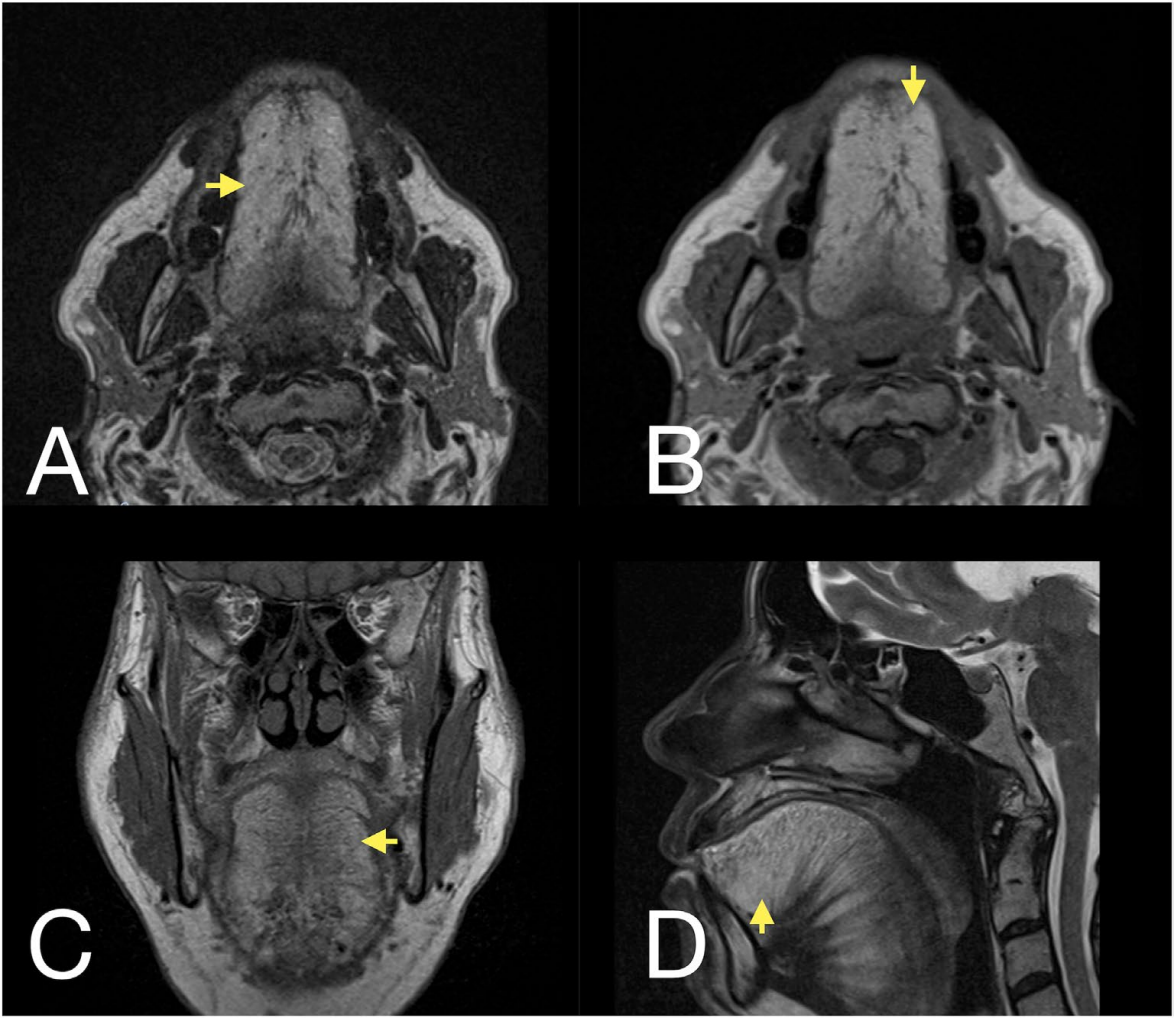
(A) Axial T2‐weighted MR image showing the tongue with a marked reduction in volume following symmetrical bilateral glossectomy, with persistent high signal intensity (arrowheads) typical of adipose tissue. (B) Axial T1‐weighted MR image showing a similar high signal intensity pattern. (C) Coronal T1‐weighted MR image confirming persistent high signal intensity (arrowheads). (D) Sagittal T2‐weighted MR image demonstrating residual benign symmetrical lipomatosis (BSL) with high signal intensity (arrowheads) in a tongue reduced by approximately 50% postoperatively.

The patient recovered well following surgery. He was hospitalized for 3 days and managed with acetaminophen and naproxen for pain control. He tolerated oral fluids by the second day of hospitalization and began eating solid food by day seven. Wound healing was adequate, with no evidence of dehiscence. At two‐ and four‐week follow‐ups, the patient reported substantial symptomatic relief, including improvement in dysarthria and a reduction in dyspnea and dysphagia (Figure 4). Histopathological examination revealed mature adipose tissue without atypia, arranged in a diffuse growth pattern with interspersed muscle fibers. No lipoblasts or immature stromal cells were identified (Figure 5). Additionally, follow‐up MRI at 6 months demonstrated a tongue with a marked postoperative volume reduction of approximately 50%, with persistent high signal intensity typical of adipose tissue, consistent with residual BSL (Figure 6).

## 5 Discussion

BSL, also known as Madelung's disease, Launois–Bensaude syndrome, or multiple symmetrical lipomatosis, is a rare disorder characterized by diffuse, nonencapsulated fat deposits predominantly affecting the neck and proximal upper limbs. This condition primarily affects middle‐aged men and is strongly associated with chronic alcohol consumption, reported in up to 90% of cases [5].

BSL has also been associated with various metabolic disorders, including hyperuricemia, hyperlipidemia, diabetes mellitus, hypertension, hepatic disease, hypothyroidism, and renal tubular acidosis [1, 2, 3]. Although most cases are linked to chronic alcohol consumption, our patient denied alcohol use, aligning with the less common, non‐alcohol‐related presentations. He did, however, have a history of hypertension. While BSL lesions are typically asymptomatic, as were the previously resected lipomatous masses in our patient's neck and shoulders, symptoms can develop in rare cases when the lesions infiltrate vital structures. In this case, the tongue involvement led to neurological symptoms and upper airway compromise.

Patients with BSL may develop severe and potentially life‐threatening complications, including mediastinal syndrome, tracheobronchial obstruction, dysphagia, dysphonia, restricted neck mobility, and both somatic and autonomic neuropathies [1]. Spontaneous regression has not been reported, and the associated decline in quality of life can be significant [3]. Surgical excision remains the definitive treatment, aiming to address both functional impairment and symptomatic burden.

Given the potential severity of these complications, including BSL in the differential diagnosis of macroglossia is crucial to facilitate early diagnosis and improve patient care. Diagnosis is based on clinical evaluation, imaging studies, and histopathology, which typically reveal deposits of normal adipose tissue [1, 4].

Early diagnosis is essential, as further lesion growth may preclude complete resection while preserving anatomical structures and functional outcomes. In this patient, the partial bilateral glossectomy removed approximately 30% of the tongue, targeting peripheral fat deposits while preserving the tip and improving functionality and aesthetics. The surgical option was chosen as the treatment since the patient exhibited symptoms that significantly affected his quality of life. His tongue was 50% larger than an average tongue, as shown in Figure 1. Additionally, the findings from the MRI were highly suggestive of a benign condition, making a confirmatory biopsy unnecessary. We decided to follow up with our patient for 4 years, as recurrence occurs in 39% of patients within 3.8 years of excision [4]. This case is significant as the first reported in Latin America, demonstrating the value of early diagnosis in a region where evidence is limited and the disease may be underreported.

## 6 Conclusion

Madelung's disease is a rare condition that is gaining recognition with increased clinical awareness, highlighting the need for timely and effective management. Surgical intervention is essential to preserve function, aesthetics, and quality of life. This case underscores the challenges of atypical presentations, such as symmetrical lipomatosis of the tongue, and reinforces the importance of timely intervention and a multidisciplinary approach.

## Author Contributions

**Daniela Salamanca‐Ramirez:** conceptualization, formal analysis, investigation, methodology, writing – original draft, writing – review and editing. **Daniel Felipe Villa‐Zuluaga:** conceptualization, methodology, writing – original draft, writing – review and editing. **Diego Alejandro Ardila‐Torres:** conceptualization, writing – original draft, writing – review and editing.

## Funding

The authors have nothing to report.

## Consent

Written informed consent for the publication of this case and associated images was obtained from the patient's legal guardian. Institutional Review Board approval was granted (IRB No. 73; approval date: September 26, 2024).

## Conflicts of Interest

The authors declare no conflicts of interest.

## Data Availability

Data sharing not applicable to this article as no datasets were generated or analysed during the current study.
